# Dexmedetomidine ameliorates high glucose-induced epithelial-mesenchymal transformation in HK-2 cells through the Cdk5/Drp1/ROS pathway: Dexmedetomidine alleviates high glucose-induced EMT

**DOI:** 10.3724/abbs.2023220

**Published:** 2023-11-27

**Authors:** Fei Wang, Weilong Xu, Xiaoge Liu, Jun Zhang

**Affiliations:** 1 Department of Anesthesiology Fudan University Shanghai Cancer Center Shanghai 200032 China; 2 Department of Oncology Shanghai Medical College Fudan University Shanghai 200032 China; 3 Department of Anesthesiology the Affiliated Hospital of Qingdao University Qingdao 266000 China

**Keywords:** dexmedetomidine, high glucose, epithelial-mesenchymal transformation, cyclin-dependent kinase 5, dynamin-related protein 1

## Abstract

Epithelial-mesenchymal transformation (EMT) plays an important role in the progression of diabetic nephropathy. Dexmedetomidine (DEX) has shown renoprotective effects against ischemic reperfusion injury; however, whether and how DEX prevents high glucose-induced EMT in renal tubular epithelial cells is incompletely known. Here, we conduct *in vitro* experiments using HK-2 cells, a human tubular epithelial cell line. Our results demonstrate that high glucose increases the expressions of EMT-related proteins, including Vimentin, Slug, Snail and Twist, while decreasing the expression of E-cadherin and increasing Cdk5 expression in HK-2 cells. Both *Cdk5* knockdown and inhibition by roscovitine increase the expressions of E-cadherin while decreasing the expressions of other EMT-related markers. DEX inhibits Cdk5 expression without affecting cell viability and changes the expressions of EMT-related markers, similar to effects of Cdk5 inhibition. Furthermore, Cdk5 is found to interact with Drp1 at the protein level and mediate the phosphorylation of Drp1. In addition, Drp1 inhibition with mdivi-1 could also restrain the high glucose-induced EMT process in HK-2 cells. Immunofluorescence results show that roscovitine, Mdivi-1 and DEX inhibit high glucose-induced intracellular ROS accumulation, while the oxidant H _2_O _2_ eliminates the protective effect of DEX on the EMT process. These results indicate that DEX mitigates high glucose-induced EMT progression in HK-2 cells via inhibition of the Cdk5/Drp1/ROS pathway.

## Introduction

Diabetic nephropathy (DN) is one of the main microvascular complications induced by diabetes mellitus [1]. Tubulointerstitial fibrosis (TIF) has been regarded as the final stage of progressive nephropathy, the degree of which is closely related to renal outcomes [2]. Recently, it has been found that epithelial-mesenchymal transformation (EMT) plays an important role in the process of TIF. EMT is a process in which epithelial cells lose their epithelial characteristics and gain mesenchymal characteristics, often manifested by decreases in the expression of E-cadherin and increases in the expressions of Vimentin, Slug, Snail, Twist and other molecules [3]. EMT is also a source of matrix-generating fibroblasts in the kidney, which plays a role in the synthesis and secretion of extracellular matrix (ECM) proteins [4]. The excessive deposition of tubulointerstitial ECM proteins leads to interstitial fibrosis, which is a typical hallmark of DN.

Cyclin-dependent kinase 5 (Cdk5) belongs to the family of cyclin-dependent kinases that phosphorylate serine and threonine residues and is involved in cell cycle regulation [5]. In kidney tissue, Cdk5 is mainly expressed in terminally differentiated glomerular cells such as podocytes. It acts as the dominant regulator of podocyte survival during glomerular disease [ 6, 7]. Nevertheless, Cdk5 is also expressed in renal tubular cells. Treatments with Cdk5 inhibitors can promote the formation of prosurvival Cdk5/cyclin1 complexes and enhance cell survival upon ischemia-reperfusion injury [8]. However, whether Cdk5 is involved in EMT is still unknown.

Dynamin-related protein 1 (Drp1) is a cytoplasmic protein that belongs to the family of large GTPases, acting as a key regulator of mitochondrial fission [9]. It has been reported that Cdk5 mediates Drp1 phosphorylation to drive mitochondrial defects, thus leading to the overproduction of reactive oxygen species (ROS) [ 10, 11], whereas intracellular ROS accumulation is a primary contributor to tubular EMT, resulting in subsequent renal fibrosis [12].

Dexmedetomidine (DEX), an alpha 2-adrenergic receptor agonist, is widely used in clinical settings as a sedative/analgesic agent. Numerous clinical and preclinical studies have shown that DEX establishes its protective effects on various important organs, including the brain, liver and lung. In terms of the kidney, DEX can stabilize renal hemodynamics and increase renal blood flow and urine output. DEX has also been reported to protect against contrast-induced kidney injury, postoperative renal injury and sepsis-induced kidney injury [ 13‒ 15], suggesting that DEX may also play a beneficial role in DN.

To date, there have been no relevant studies on the effects of DEX in DN. Therefore, we performed this *in vitro* study to investigate whether and how DEX alleviates EMT progression using a high-glucose-induced human tubular cell model. This will lay a research foundation for us to further study the effect of DEX on DN.

## Materials and Methods

### Cell culture and treatments

Human tubular epithelial cell lines (HK-2) were purchased from American Type Culture Collection (ATCC; Manassas, USA). The cells were cultured in three different DMEM groups. In the normal-glucose group, the cells were cultured in DMEM with 5 mM glucose for 6 days. In the high-glucose group, the cells were cultured in DMEM with 25 mM glucose. In contrast, glucose (5 mM) plus mannitol (20 mM) was used at the same time to eliminate the possible influence of osmotic pressure.

To further investigate the role of Cdk5 in the EMT process in HK2 cells, roscovitine, a Cdk5 inhibitor, was applied in our experiments. After the HK-2 cells were cultured in DMEM with 5 mM glucose or cultured in DMEM with 25 mM glucose for three days, roscovitine (15 μmol) was added to the medium and incubated for 24 h. To further investigate the association between Drp1 phosphorylation and EMT, the Drp1 inhibitor Mdivi-1 was used to inhibit the phosphorylation of Drp1. To validate whether DEX inhibits high glucose-induced EMT progression through decreasing oxidative stress, H _2_O _2_ (500 μM) was added to high glucose DMEM and incubated with HK-2 cells for 4 h after DEX treatment.

### Western blot analysis

After culture, HK-2 cells were washed with PBS and then lysed in RIPA lysis buffer supplemented with PMSF (Beyotime Biotechnology, Shanghai, China). The protein concentration of the cell extract was measured using a BCA protein assay kit (Epizyme, Shanghai, China). The same amount of protein from different groups, which was approximately 50 μg, was separated by sodium dodecyl sulfate-polyacrylamide gel electrophoresis (SDS-PAGE). The separated proteins were then transferred to PVDF membranes (Millipore, Billerica, USA) and incubated with the primary antibodies. The antibodies used in the present study were as follows: polyclonal antibodies against GAPDH (1:1000, 10494-1-AP; ProteinTech, Wuhan, China), Cdk5 (1:2000, AB40773; Abcam, Cambridge, UK), Drp1 (1:1000, 12957-1-AP; ProteinTech), p-Drp1 (Phospho-Ser616) (1:1000, #12749; Sabbiotech, Greenbelt, USA,), Twist (1:1000, 20465-1-AP; ProteinTech), Slug (1:1000, 12129-1-AP; ProteinTech), Snail (1:1000, A5243; ABclonal, Wuhan, China) Vimentin (1:1000, 10366-1-AP; ProteinTech) and E-cadherin (1:1000, 20874-1-AP; ProteinTech). After incubation with primary antibodies, membranes were incubated with a HRP-conjugated secondary antibody (#7074; Cell Signaling Technology, Danvers, USA). Finally, blots were visualized by using the Tanon-5200 Chemiluminescent imaging system (Shanghai, China).

### Quantitative real-time PCR (qRT-PCR)

Total RNA from HK-2 cells was extracted by Trizol (Invitrogen, Grand Island, USA), and then complementary DNA (cDNA) was synthesized through reverse transcription. Real-time PCR was performed using Hieff® qPCR SYBR Green Master Mix (Yeasen, Shanghai, China) on an ABI7500 Real-Time PCR system (Applied Biosystems, Foster City, USA). All transcript levels were compared using the relative values of *GAPDH* levels as the reference. The primers used in the present study are shown in Table 1.

**Table:** **Table 1** Sequences of primers used for real-time RT-PCR analysis

Gene	Primer sequence (5′→3′)
*Cdk5*	F: GATGATGAGGGTGTGCCGAGTTC
	R: TGAAGCCTGACGATGTTCTTGTGC
*E-cadherin*	F: GCCATCGCTTACACCATCCTCAG
	R: CTCTCTCGGTCCAGCCCAGTG
*Vimentin*	F: TGAATGACCGCTTCGCCAACTAC
	R: CTCCCGCATCTCCTCCTCGTAG
*Slug*	F: ACTGTGTGGACTACCGCTGCTC
	R: GGAGGAGGTGTCAGATGGAGGAG
*Snail*	F: CCTCGCTGCCAATGCTCATCTG
	R: GCTCTGCCACCCTGGGACTC
*Twist*	F: CCATCCTCACACCTCTGCATTCTG
	R: GGCTGATTGGCACGACCTCTTG
*Drp1*	F: TCACCCGGAGACCTCTCATTC
	R: GGTTCAGGGCTTACTCCCTTAT
*GAPDH*	F: GGAGCGAGATCCCTCCAAAAT
	R: GGCTGTTGTCATACTTCTCATGG

### Small RNA interference (siRNA)

For RNA interference, HK-2 cells were transfected with siRNA using Lipofectamine 3000 (Invitrogen) according to the manufacturer’s instructions. To silence *Cdk5*, two different siRNAs targeting Cdk5 were used: siRNA-a, 5′-UGACCAAGCUGCCAGACUA-3′, and siRNA-b, 5′-UCGUCAGGCUUCAUGACGU-3′. The negative control is shown as follows: 5′-UUCUCCGAACGUGUCACGUdTdT-3′.

### Coimmunoprecipitation (Co-IP)

A certain number of HK-2 cells were seeded in 100-mm cell culture dishes and cultured for approximately 2‒3 days. When they were 90%‒100% complete, the cells were harvested using disposable cell scrapers and extracted with cell lysis buffer (Cell Signaling Technology) to obtain protein lysates. The lysate supernatant (30 μL) was transferred to an Eppendorf tube as the input. The other supernatant was incubated with the corresponding primary antibodies and A/G Dynabeads (Thermo Fisher, Waltham, USA) sequentially at 4°C overnight to obtain endogenous IP. The input, IgG and IP fractions were analyzed by western blot analysis.

### Cell viability assay

The cell viability of HK-2 cells was detected using a commercially available Cell Counting Kit-8 (CCK-8) viability assay kit (Yeasen). The cells were seeded into 96-well plates, each well with 200 μL DMEM, and then varying concentration gradients of dexmedetomidine were added to each well. Then, 20 μL CCK-8 was added to each well at different time points, such as 24 h, 48 h, 72 h and 96 h. Absorbance was measured at 490 nm using an Infinite M200 Microplate Reader (Tecan, Männedorf, Switzerland) to reflect the cell viability.

### Radical oxygen species (ROS) measurements

The ROS levels in HK-2 cells were assayed using a commercially available ROS assay kit (Beyotime Biotechnology). The cell permeable reagent DCFH-DA was used to detect intracellular ROS production, while DAPI was applied to indicate the nuclei of living cells. HK-2 cells in different groups seeded in 96-well plates were washed twice, and then the culture media were replaced by DMEM containing 25 μM DCFH-DA. After the cells were incubated in the dark for 30 min, DCFH-DA fluorescence was measured at 488 nm of exciting light (BioTek Instruments, Winooski, USA) in a fluorescence microscope (Leica, Wetzlar, Germany).

### Statistical analysis

Data are presented as the mean±SD acquired from at least five experiments performed separately. The significant differences between two groups were analyzed using Student’s *t* test, while the comparisons among groups were analyzed using one-way analysis of variance (ANOVA). All statistical analyses were performed using GraphPad Prism 8.0 software (GraphPad Software, San Diego, USA). *P* value less than 0.05 was considered statistically significant.

## Results

### DEX reverses high glucose-induced EMT progression in HK-2 cells

**Figure 1:**
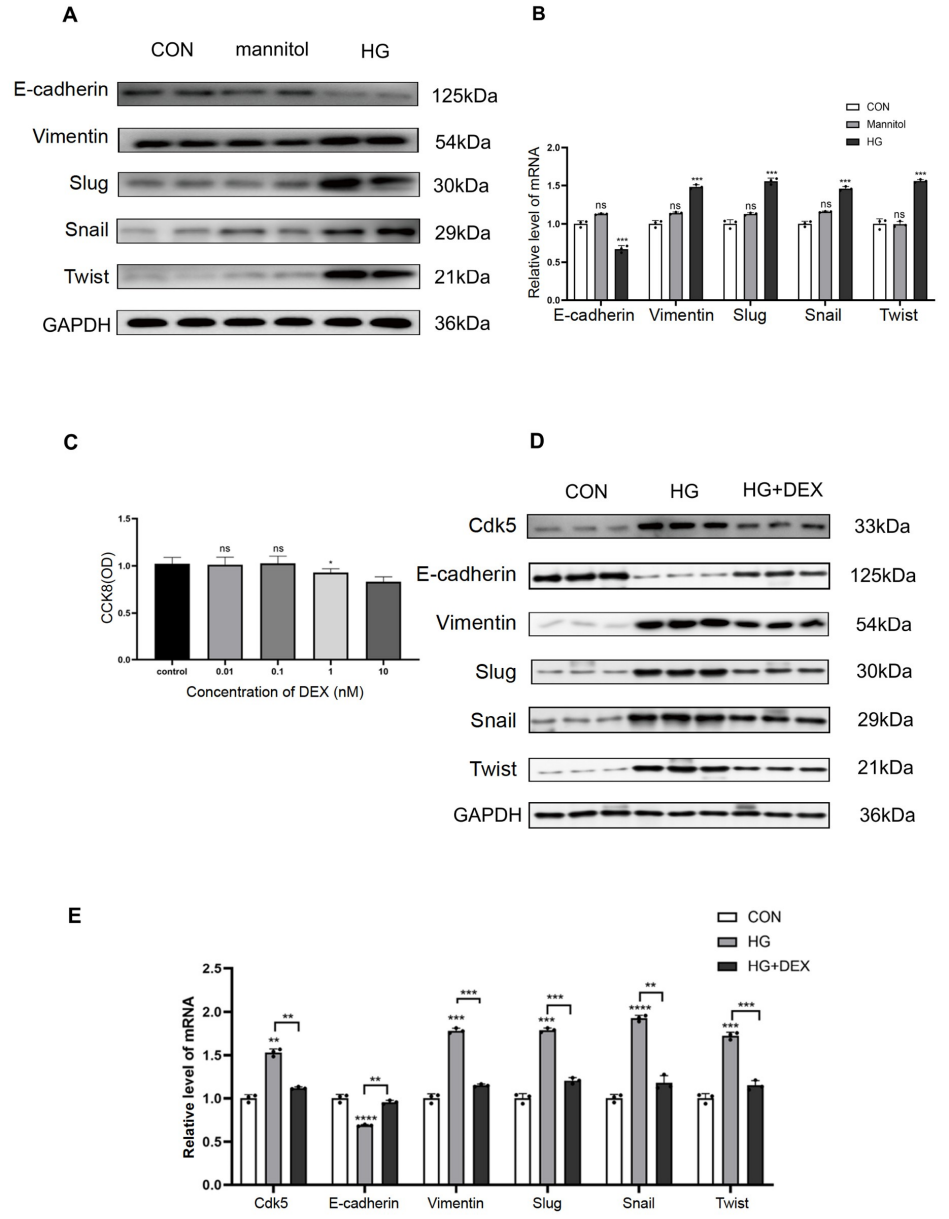
Figure 1 Dexmedetomidine reverses changes in high glucose-induced EMT markers (A) Western blot analysis of E-cadherin, Vimentin, Slug, Snail and Twist in HK-2 cells in DMEM with different concentrations of glucose. (B) qPCR analysis of mRNA expressions of E-cadherin, Vimentin, Slug, Snail and Twist in HK-2 cells. (C) CCK8 assay of HK-2 cells exposed to different dexmedetomidine concentrations. When the concentration was 1 nM or higher, the survival of cells was decreased. When the concentration was 0.1 nM or less, survival was not affected. (D) Western blot analysis of the expressions of Cdk5, E-cadherin, Vimentin, Slug, Snail and Twist in HK-2 cells exposed to 0.1 nM dexmedetomidine. (E) PCR analysis of the mRNA expressions of Cdk5, E-cadherin, Vimentin, Slug, Snail and Twist in HK-2 cells. *P<0.05, **P<0.01, ***P<0.001, and ****P<0.0001, n=5/group. CON, control; HG, high glucose.

To explore whether high glucose levels regulate the EMT process in HK-2 cells, we measured the expressions of EMT-related markers in different culture media. We found that the expression of epithelial marker E-cadherin was decreased, while the expressions of mesenchymal markers Vimentin, Slug, Snail and Twist were increased at both the protein and mRNA levels after high glucose stimulation ( Figure 1A,B). However, mannitol did not change the expressions of these EMT markers in HK-2 cells, suggesting that high glucose rather than hyperosmolality promotes the EMT process in HK-2 cells.

Meanwhile, the CCK-8 experiment showed that DEX at a concentration of 1 nM or more decreased cell survival, while a concentration of 0.1 nM or less did not affect survival ( Figure 1C). Therefore, 0.1 nM DEX was selected for *in vitro* intervention. DEX showed a protective effect on high glucose-induced EMT of HK-2 cells, as demonstrated by lower expressions of Vimentin, Slug, Snail, and Twist and higher expression of E-cadherin ( Figure 1D,E). Furthermore, DEX also reversed high glucose-induced Cdk5 overexpression in HK-2 cells ( Figure 1D,E).

### Cdk5 is involved in high glucose-induced EMT in HK-2 cells

**Figure 2:**
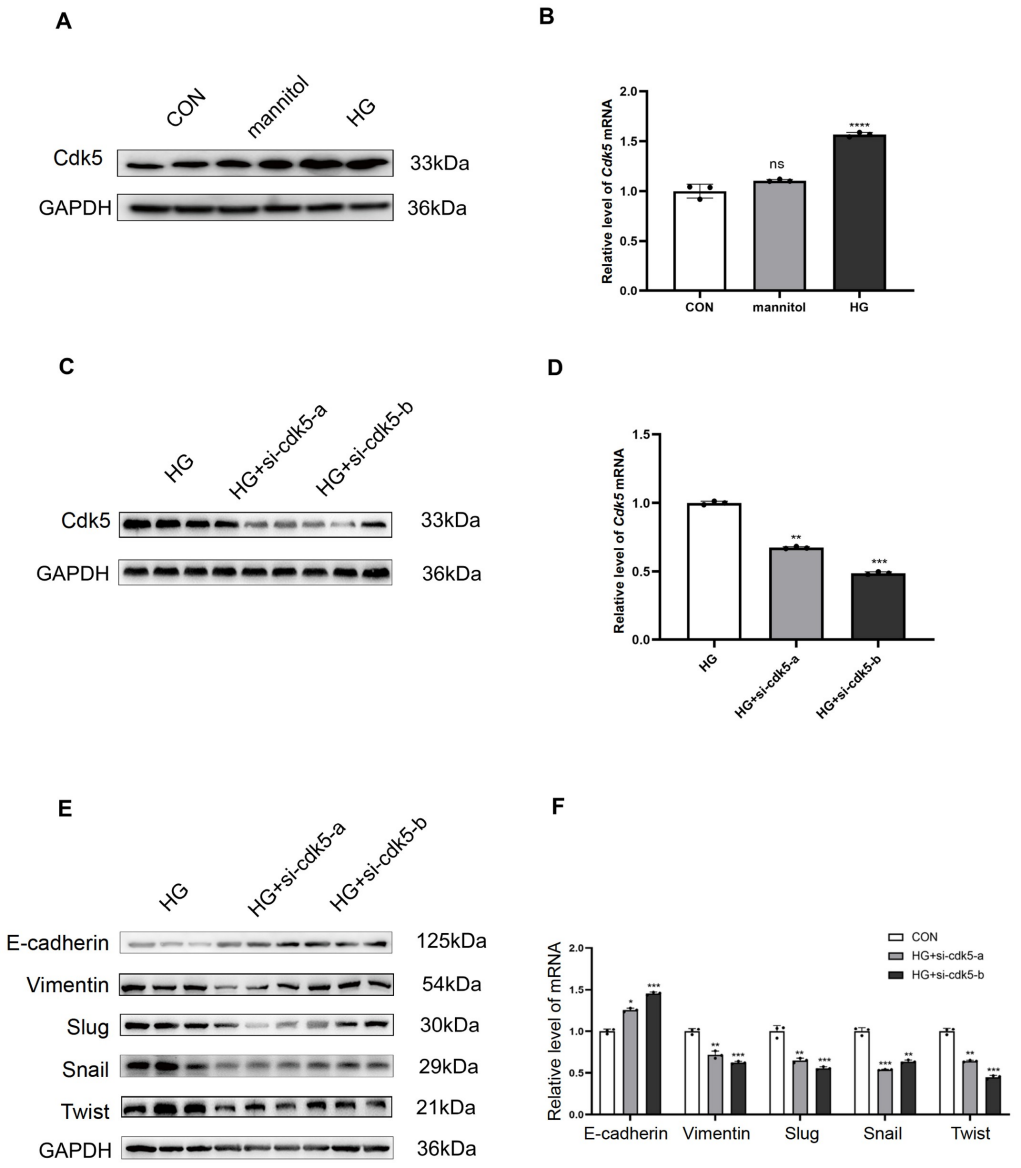
Figure 2 Dexmedetomidine and *Cdk5* silencing reversed the high glucose-stimulated EMT process (A) Western blot analysis of Cdk5 expression in HK-2 cells exposed to different glucose concentrations in DMEM. (B) PCR analysis of Cdk5 mRNA expression in HK-2 cells. (C) Western blot analysis of Cdk5 expression in HK-2 cells after si-Cdk5 treatment. (D) PCR analysis of Cdk5 mRNA expression in HK-2 cells. (E) Western blot analysis of E-cadherin, Vimentin, Slug, Snail and Twist expressions in HK-2 cells. (F) PCR analysis of E-cadherin, Vimentin, Slug, Snail and Twist mRNA expressions in HK-2 cells. *P<0.05, **P<0.01, ***P<0.001, and ****P<0.0001, n=5/group. CON, control; HG, high glucose.

To examine whether high glucose promotes the EMT process through Cdk5 in HK-2 cells, we measured Cdk5 expression after different treatments. We found that the expression of Cdk5 was increased at both the protein and mRNA levels in high-glucose DMEM ( Figure 2A,B). Furthermore, two independent siRNAs against Cdk5 were used to downregulate Cdk5 expression, and their effects were confirmed by western blot analysis ( Figure 2C) and qRT-PCR ( Figure 2D). The results showed that knockdown of *Cdk5* significantly inhibited high glucose-induced increases in Vimentin, Slug, Snail and Twist levels but elevated E-cadherin levels in HK-2 cells ( Figure 2E,F), suggesting that silencing of *Cdk5* eliminates high glucose-induced EMT progression in HK-2 cells.

### Inhibition of Cdk5 restrains high glucose-induced EMT

**Figure 3:**
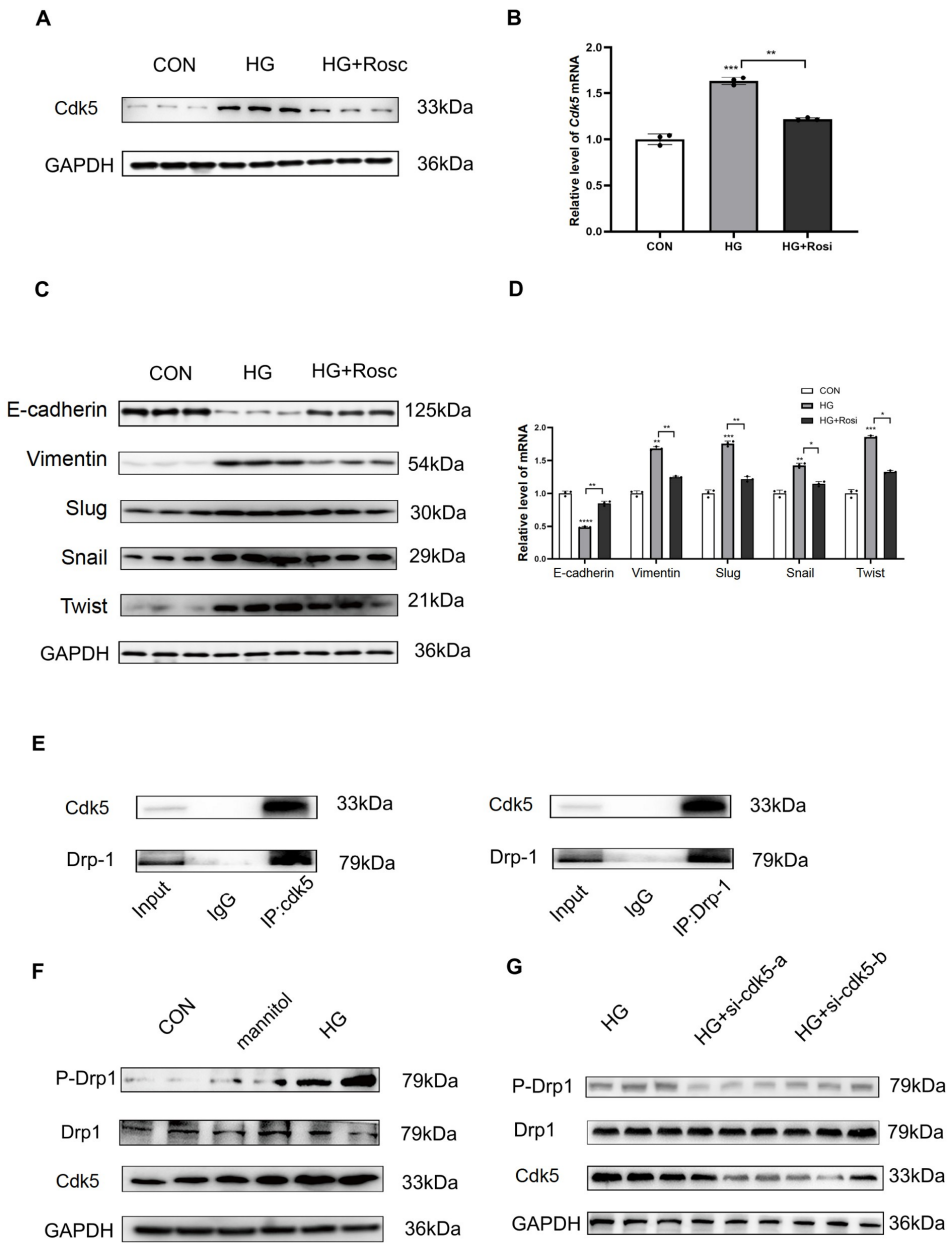
Figure 3 Inhibition of Cdk5 reverses EMT marker expression induced by high glucose, which increases Drp1 phosphorylation in HK-2 cells (A) Western blot analysis of Cdk5 expression in HK-2 cells treated with roscovitine (Rosi). (B) PCR analysis of Cdk5 mRNA expression in HK-2 cells. (C) Western blot analysis of E-cadherin, Vimentin, Slug, Snail and Twist expressions in HK-2 cells. (D) PCR analysis of E-cadherin, Vimentin, Slug, Snail and Twist mRNA expressions in HK-2 cells. (E) Co-IP verified the interaction between Cdk5 and Drp1 in HK-2 cells. (F,G) Western blot analysis of Cdk5, Drp1 and p-Drp-1 expressions in different treatment groups. *P<0.05, **P<0.01, ***P<0.001, ****P<0.0001, n=5/group. CON, control; HG, high glucose.

To further validate the role of Cdk5 in EMT progression, the Cdk inhibitor roscovitine was used to treat HK-2 cells. The results showed that roscovitine decreased Cdk5 expression at both the protein and mRNA levels in HK-2 cells ( Figure 3A,B). Roscovitine reversed the changes in the expressions of EMT-related proteins, as evidenced by the decrease of high glucose-induced elevated Vimentin, Slug, Snail, and Twist levels and the increase of E-cadherin level in HK-2 cells ( Figure 3C,D). The EMT improvement by both deletion and inhibition of Cdk5 confirms that Cdk5 plays an important role in DEX-mediated reversal of EMT progression.

Furthermore, Co-IP experiment showed that Cdk5 interacted with Drp1 at the protein level ( Figure 3E). The level of Drp1 phosphorylation was increased along with elevated Cdk5 expression elicited by high glucose. However, the expression of Drp1 itself showed only a small change ( Figure 3F). Similarly, *Cdk5* knockdown also inhibited Drp1 phosphorylation without changing Drp1 expression ( Figure 3G). These results imply that Cdk5 promotes high glucose-induced EMT progression in HK-2 cells, which may be mediated by Drp1 phosphorylation.

### DEX restrains high glucose-induced EMT through inhibition of Drp1 phosphorylation

**Figure 4:**
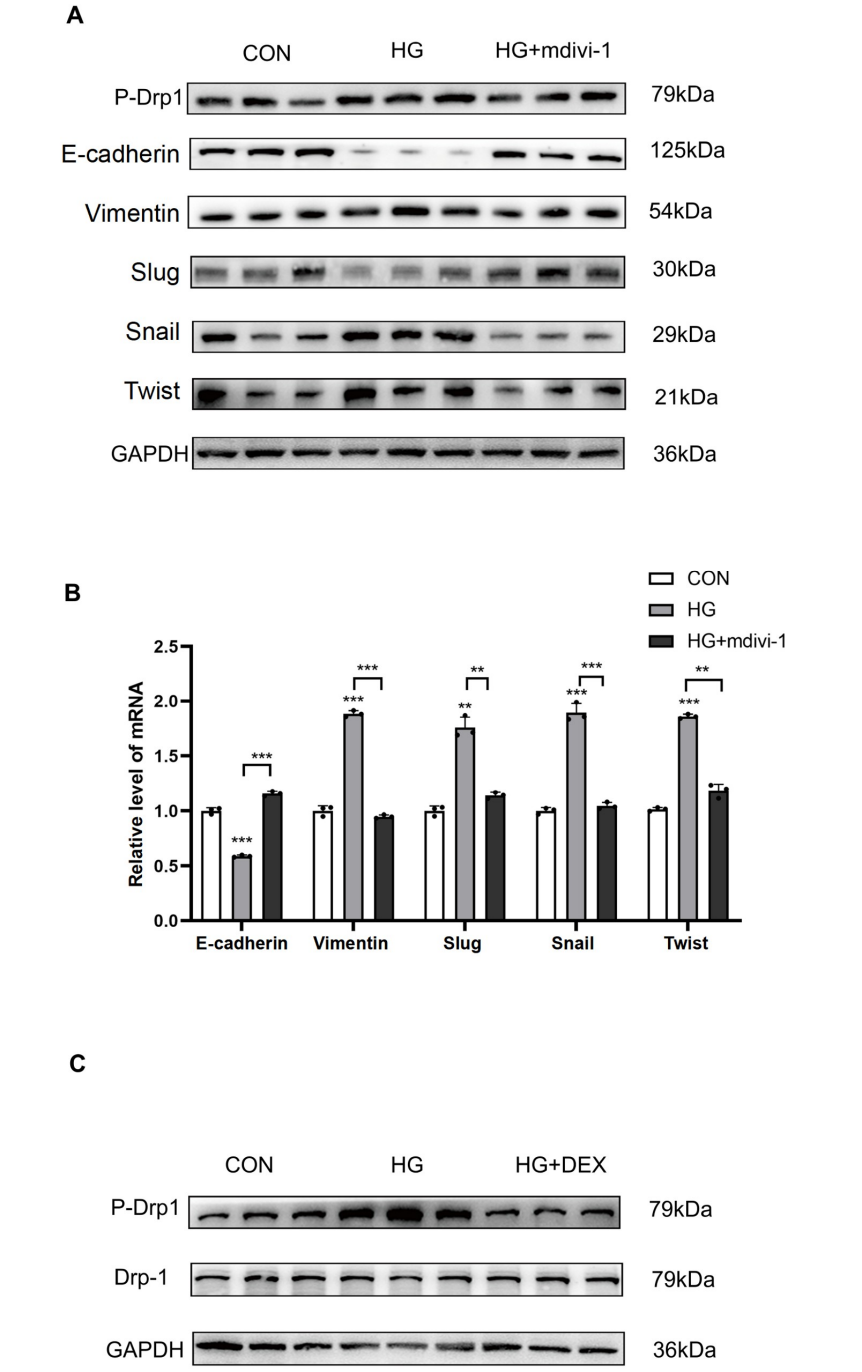
Figure 4 The role of Drp1 phosphorylation in dexmedetomidine protection against high glucose-induced EMT in HK-2 cells (A) Western blot analysis of the expressions of p-Drp1, E-cadherin, Vimentin, Slug, Snail and Twist in HK-2 cells treated with Mdivi-1. (B) PCR analysis of the mRNA expressions of E-cadherin, Vimentin, Slug, Snail and Twist in HK-2 cells. (C) Western blot analysis of the expressions of p-Drp1 and Drp1 in HK-2 cells treated with dexmedetomidine. *P<0.05, **P<0.01, ***P<0.001, and ****P<0.0001, n=5/group. CON, control; HG, high glucose.

To further clarify the link between Drp1 phosphorylation and EMT, a Drp1 inhibitor, Mdivi-1, was used to inhibit Drp1 phosphorylation. We found that Mdivi-1 reversed the high glucose-induced increase in Drp1 phosphorylation and reduced the expressions of Vimentin, Slug, Snail, and Twist and increased the expression of E-cadherin in HK-2 cells ( Figure 4A,B). Similarly, DEX treatment also inhibited Drp1 phosphorylation ( Figure 4C), further suggesting that DEX reduces EMT progression via Cdk5-mediated Drp1 phosphorylation.

### DEX prevents high glucose-induced EMT progression through the Cdk5/Drp1/ROS pathway

**Figure 5:**
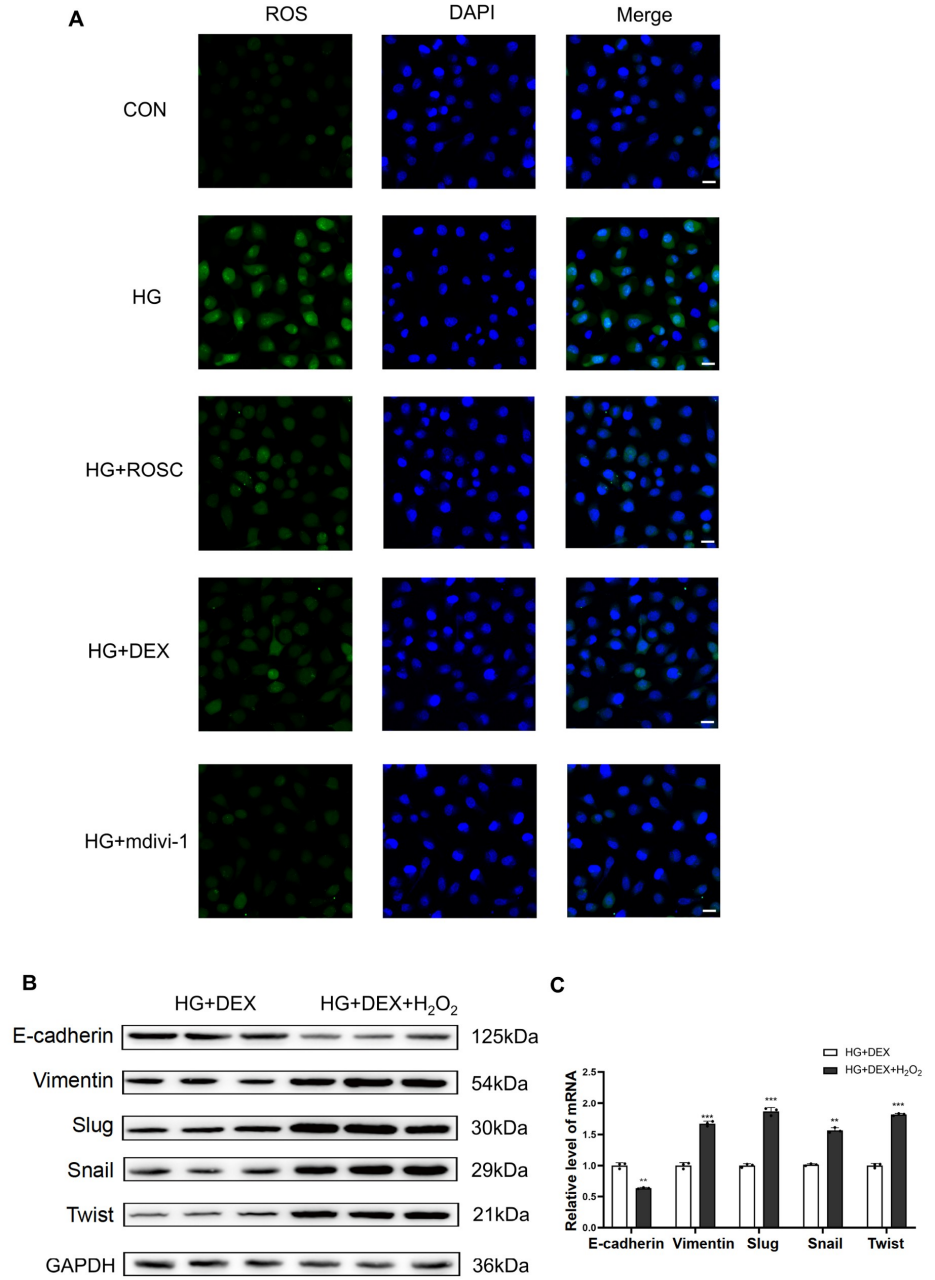
Figure 5 Roscovitine, Mdivi-1 and dexmedetomidine can inhibit ROS accumulation in high glucose-treated HK-2 cells (A) Representative immunofluorescence images indicating the ROS production of HK-2 cells in different groups (scale bar: 25 μm). (B) Western blot analysis for expressions of E-cadherin, Vimentin, Slug, Snail and Twist. (C) PCR analysis of mRNA expressions of E-cadherin, Vimentin, Slug, Snail and Twist in DEX-treated HK-2 cells with or without H2O2 stimulation. *P<0.05, **P<0.01, ***P<0.001, and ****P< 0.0001, n=5/group. CON, control; HG, high glucose.

We found that high glucose significantly increased ROS production, while DEX reduced the accumulation of intracellular ROS in HK-2 cells. ROS overproduction is associated with high glucose-triggered activation of the EMT process [16]. Therefore, to investigate whether DEX alleviates ROS-induced EMT via Cdk5-Drp1 signaling, inhibitors of Cdk5 and Drp1 were used in high glucose-treated HK-2 cells. The results were similar to the findings of DEX treatment; both roscovitine and Mdivi-1 inhibited ROS production to varying degrees ( Figure 5A), suggesting that inhibition of oxidative stress through the Cdk5-Drp1 pathway may underlie the protective effects of DEX on high glucose-induced EMT progression. To further test this finding, HK-2 cells in DMEM with high glucose were stimulated by H _2_O _2_ for 4 h after DEX treatment. Our results showed that compared with DEX-treated HK-2 cells in high glucose DMEM, the expression of epithelial marker E-cadherin was decreased, while the expressions of mesenchymal markers Vimentin, Slug, Snail and Twist were increased again at both the protein and gene levels after stimulation with H _2_O _2_ ( Figure 5B,C). These data indicate that DEX prevents high glucose-induced EMT progression through the Cdk5/Drp1/ROS pathway.

## Discussion

EMT plays an important role in the interstitial fibrosis of DN, which is the major reason for renal dysfunction [ 17, 18]. Consistent with previous findings [ 19, 20], the present study showed that high glucose promoted the EMT process in human primary tubular cell cultures. Furthermore, our results showed that high glucose increased Cdk5 expression, Drp1 phosphorylation and ROS overproduction, leading to EMT progression in HK-2 cells, while DEX inhibited the high glucose-induced EMT process by inhibiting the Cdk5/Drp1/ROS pathway. This provides a potential therapeutic target for DN.

Roscovitine is a purine analog that inhibits the activity of cyclin-dependent kinases, including Cdk1, Cdk2 and Cdk5 [21]. It has been found that Cdk5 inhibition by roscovitine could ameliorate insulin resistance and increase glucose uptake in neuronal cells [22]. In our study, roscovitine also restrained the EMT process in HK-2 cells. All of these results suggest that Cdk5 is indeed involved in the EMT process of HK-2 cells. Most previous studies focused on the protective effects of DEX against intraoperative acute kidney injury [ 23‒ 25]. Few studies have focused on the effects of DEX on chronic kidney diseases such as DN. Our study demonstrated that DEX at concentrations that do not affect cell viability could also suppress EMT progression by reversing Cdk5-mediated expressions of EMT-related markers.

What is the key factor that links Cdk5 to EMT? The answer is not clearly known. Cdk5 was reported to promote Drp1 phosphorylation to drive mitochondrial defects, thus leading to ROS overproduction [10]. A previous study also demonstrated that inhibition of Cdk5 could attenuate cognitive deficits induced by chronic exposure to ethanol by inhibiting Drp1 phosphorylation at S616 [26]. In the present study, we found that Cdk5 and Drp1 had protein-protein interaction. The expression of Cdk5 is closely associated with Drp1 phosphorylation. From these results, we found that Cdk5 mainly affected the phosphorylation of Drp1 but had little effect on the total expression of Drp1 in cells. We hypothesize that intracellular Drp1 is inactive and only active when phosphorylated to P-Drp1, thus promoting EMT changes in cells. Downregulating the expression of Cdk5 by si-Cdk5s also influenced the phosphorylation of Drp1 but had little effect on the total expression of Drp1 in cells. Genetic manipulation further verified their interaction during high glucose exposure, which can promote Drp1-dependent mitochondrial fission and decrease mitochondrial fusion, leading to mitochondrial dysfunction.

Mitochondrial dysfunction is involved in the development of diabetic cardiomyopathy [27]. Drp1 regulates mitochondrial fission by changing its phosphorylation level. Mdivi-1 is a putative small-molecule inhibitor of mitochondrial fission that specifically targets Drp1 [ 28, 29]. Studies have reported that Mdivi-1 can reversibly inhibit mitochondrial complex I and reduce ROS generation in cells and tissues [30]. In our study, we found that Mdivi-1 can also restrain the EMT process in HK-2 cells. Collectively, these data suggest that DEX mitigates high glucose-induced EMT progression by protecting mitochondrial function.

Evidence has shown that ROS can induce EMT progression through different pathways [ 31‒ 33]. Some antioxidants could attenuate EMT progression by decreasing ROS production [ 34, 35]. As previously indicated, we also found that high glucose significantly increased intracellular ROS accumulation in HK-2 cells. DEX decreases high glucose-induced ROS overproduction, similar to the findings in roscovitine- and Mdivi-1-treated HK-2 cells, suggesting an antioxidative role of DEX and its renoprotective effects. Therefore, DEX inhibited high glucose-induced EMT mediated by the Cdk5/Drp1/ROS pathway, which provides a potential novel target to decelerate the progression of DN.

Nevertheless, there are still some limitations in our study. First, our results were only validated in an *in vitro* model, and an animal DN model should be used for further investigation. Second, we chose only some representative markers, such as E-cadherin, Vimentin, Slug, Snail and Twist, to study the effects of high glucose on the EMT process, and the exact mechanism underlying high glucose-induced EMT progression is still not well known. Third, our *in vitro* findings cannot be simply translated into clinical practice; however, they may provide a molecular basis for future studies.

**Figure 6:**
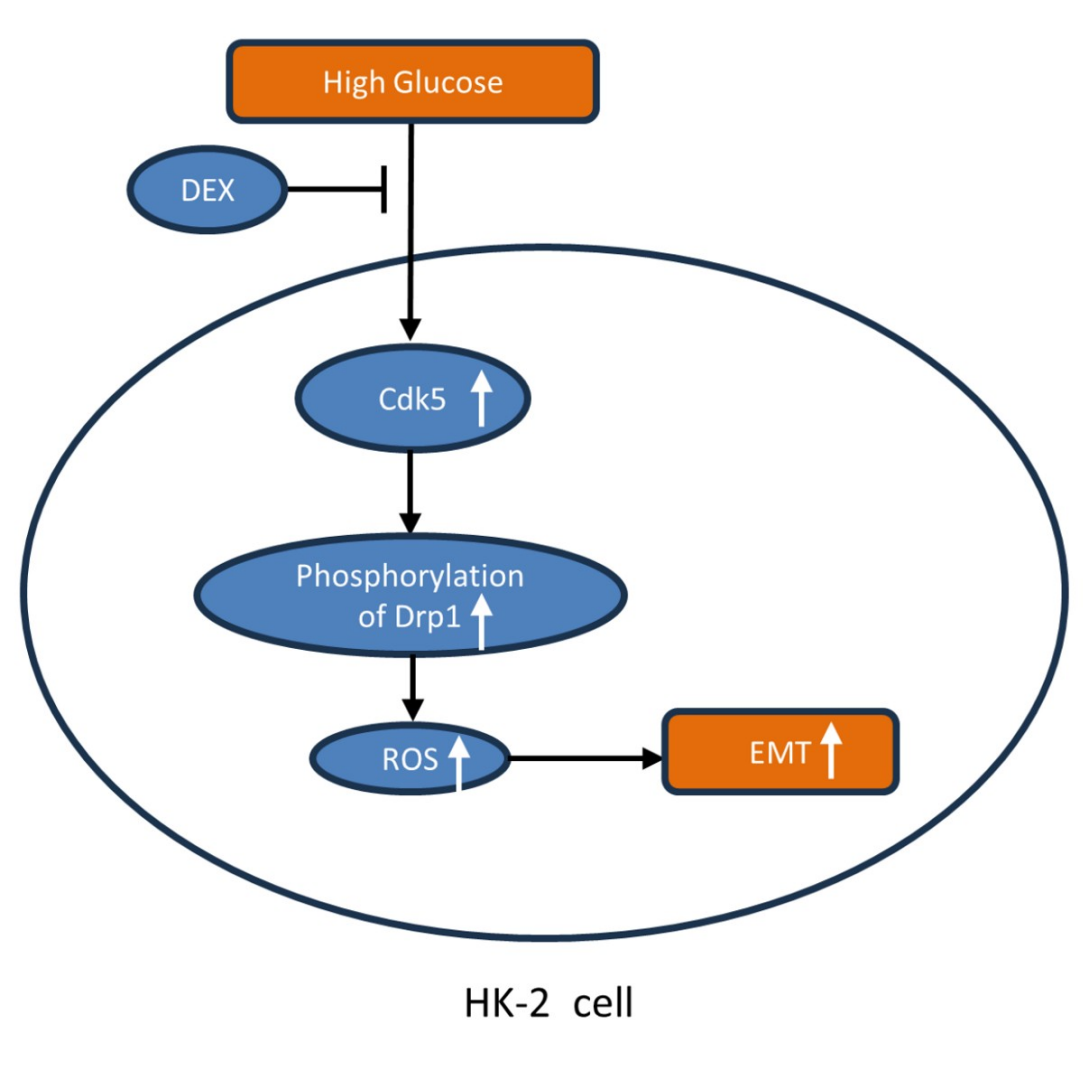
Figure 6 Dexmedetomidine ameliorates high glucose-induced EMT through the Cdk5/Drp1/ROS pathway High glucose could increase Cdk5 expression and Drp1 phosphorylation in renal tubular epithelial cells, promoting their EMT process. In contrast, DEX prevents high glucose-induced EMT progression through the Cdk5/Drp1/ROS pathway

In conclusion, high glucose could increase Cdk5 expression and Drp1 phosphorylation in renal tubular epithelial cells, promoting their EMT process. In contrast, DEX prevents high glucose-induced EMT progression through the Cdk5/Drp1/ROS pathway ( Figure 6). Since DEX is commonly used for sedation and anesthetic sparing, similar to the results from other intravenous anesthetics that prevent high glucose-induced harmful effects [36], our findings may have translational potential for diabetic patients during perioperative care.
